# Adult Onset of Acute Disseminated Encephalomyelitis (ADEM) With Associated Myelin Oligodendrocyte Glycoprotein (MOG) Antibody

**DOI:** 10.1155/crii/8603136

**Published:** 2026-04-18

**Authors:** Daniel Wilmot, Kaylee Sorrells, Sydnee Guthrie, Sapna Vyas, Mackenzie Lupov

**Affiliations:** ^1^ Department of Internal Medicine, Indiana University School of Medicine, Bloomington, Indiana, USA, indiana.edu

## Abstract

ADEM is an inflammatory and demyelinating autoimmune disorder of the central nervous system (CNS). One recognized cause of ADEM is myelin oligodendrocyte glycoprotein antibody‐associated disease (MOGAD). MOGAD affects approximately one in 10,000 people worldwide and is more prevalent in children. Patients typically present with optic neuritis, ADEM, and transverse myelitis. Many patients will experience relapses of demyelinating attacks, and thus, both acute and maintenance therapies are typically used to manage the disease. This case considers an adult female who presented with neurological deficits as a result of MOG–associated ADEM. She had minimal response to traditional therapies but responded well to treatment with IV immunoglobulin (IVIG). This case report explores her clinical course as well as unique clinical significance and conclusions due to the nontraditional use of IVIG.

## 1. Case Summary

A 46‐year‐old woman with no significant PMH presented with several days of progressive altered mental status and speech arrest. An MRI revealed multifocal demyelination of the central nervous system (CNS), and EEG showed encephalopathy without epileptiform discharges. After negative lab workup for viral and bacterial diseases, she was found to have positive MOG antibodies [1, 2]. High‐dose IV steroids were initiated, but her mental status showed no improvement. She subsequently completed seven sessions of plasma exchange, but given her continued neurological deficits, the care team elected to try IV immunoglobulin (IVIG). Prior to discharge, she was following simple commands, walking, and improving in responsiveness, although remained nonverbal and required a feeding tube. After a stay of 26 days, she was discharged to rehab on daily prednisone 50 mg, with plans to slowly taper off over 2 months. At a subsequent ER visit for a clogged PEG tube, she was able to speak and showed signs of improved swallowing and recovery in mental status. She was discharged back to rehab facility and not seen after that point, so extent of further recovery is unknown [3, 4].

## 2. Clinical Significance

This case highlights an unusual adult presentation of myelin oligodendrocyte glycoprotein antibody‐associated disease (MOGAD) without the typical optic neuritis or transverse myelitis. Previously reported adult MOGAD cases show a higher incidence in males, with a median age at onset in the mid to late 20s. [5–7] This patient’s demographics are very atypical for this condition and contribute to the intrigue of the case. This case also demonstrates the potential effectiveness of early IVIG in combination with steroids and plasmapheresis in reducing morbidity. High‐dose steroids are the first‐line treatment for acute MOGAD attacks, but IVIG is frequently used in autoimmune conditions to treat patients refractory to initial steroid therapy. The mechanisms of IVIG treatment are not fully understood, but it is generally thought to help by neutralizing autoantibodies, increasing anti‐inflammatory cytokines, and reducing overall inflammation. [8] Most studies analyzing treatment response to IVIG have been done in pediatric populations, although there is documented benefit for preventing relapse in adult patients. [4] Typical recovery time is 4–6 weeks, with further recovery after that point likely minimal. This corresponds with duration of steroid use after discharge. Follow‐up in adult‐onset MOGAD ADEM is highly variable, with many of these patients unfortunately experiencing at least one relapse [9–11]. Use of maintenance IVIG during treatment appears to significantly reduce the risk of relapse in adults with MOGAD. This likely improves long‐term neurological outcomes by preventing the disability accumulation that accompanies recurrent relapses [12, 13]. The response to IVIG in this patient adds to the growing evidence that IVIG is an effective therapy for acute treatment in adult MOGAD patients who are refractory to initial steroid therapy.

## 3. Conclusion

This atypical presentation of MOGAD underscores the importance of elucidating its pathophysiology and developing treatment strategies for patients who do not respond to standard therapies. IVIG represents a promising therapeutic option for refractory MOGAD. Future research should be directed towards elucidating factors that influence response to IVIG, early signs to indicate MOGAD testing, and potential for more direct therapy for this condition in the future.

## Funding

No funding was received for this manuscript.

## Conflicts of Interest

The authors declare no conflicts of interest.

## Data Availability

Data sharing is not applicable to this article as no datasets were generated or analyzed during the current study.

## References

[1] Sechi E. , Cacciaguerra L. , and Chen J. J. , et al.Myelin Oligodendrocyte Glycoprotein Antibody-Associated Disease (MOGAD): A Review of Clinical and MRI Features, Diagnosis, and Management, Frontiers in Neurology. (2022) 13, 10.3389/fneur.2022.885218, 885218. 35785363 PMC9247462

[2] Marsool M. D. M. , Prajjwal P. , Inban P. , Marsool A. D. M. , Tariq H. , and Hussin O. A. , Adult-Onset Acute Disseminated Encephalomyelitis: A Rare Case Report in a 26-Year-Old Female and Review of Literature, Annals of Medicine & Surgery. (2023) 85, no. 10, 5242–5245, 10.1097/ms9.0000000000001239. 37811035 PMC10553152

[3] Steriade C. , Acute Disseminated Encephalomyelitis (ADEM) in Adults, Dynamedex, 2024, https://www.dynamedex.com/condition/acute-disseminated-encephalomyelitis-adem-in-adults.

[4] Chen J. J. , Huda S. , and Hacohen Y. , et al.Association of Maintenance Intravenous Immunoglobulin With Prevention of Relapse in Adult Myelin Oligodendrocyte Glycoprotein Antibody–Associated Disease, JAMA Neurology. (2022) 79, no. 5, 518–525, 10.1001/jamaneurol.2022.0489. 35377395 PMC8981066

[5] Cacciaguerra L. , Sechi E. , and Komla-Soukha I. , et al.MOG Antibody-Associated Disease Epidemiology in Olmsted County, USA, and Martinique, Journal of Neurology. (2025) 272, no. 2, 10.1007/s00415-024-12861-9, 118. 39812824 PMC12168146

[6] Sun W. , Xie Y. , and Han A. , et al.Clinical Characteristics and Factors Associated With Recurrence and Long-Term Prognosis in Patients With MOGAD, Frontiers in Immunology. (2025) 16, 10.3389/fimmu.2025.1535571, 1535571. 40406135 PMC12095161

[7] Takai Y. , Misu T. , and Kaneko K. , et al.Myelin Oligodendrocyte Glycoprotein Antibody-Associated Disease: An Immunopathological Study, Brain. (2020) 143, no. 5, 1431–1446, 10.1093/brain/awaa102. 32412053

[8] Gelfand E. W. , Intravenous Immune Globulin in Autoimmune and Inflammatory Diseases, New England Journal of Medicine. (2012) 367, no. 21, 2015–2025, 10.1056/NEJMra1009433, 2-s2.0-84869431305. 23171098

[9] López-Chiriboga A. S. , Majed M. , and Fryer J. , et al.Association of MOG-IgG Serostatus With Relapse After Acute Disseminated Encephalomyelitis and Proposed Diagnostic Criteria for MOG-IgG–Associated Disorders, Jama Neurology. (2018) 75, no. 11, 1355–1363, 10.1001/jamaneurol.2018.1814, 2-s2.0-85055700174. 30014148 PMC6248120

[10] Misu T. , Myelin Oligodendrocyte Glycoprotein Antibody-Associated Disease: Pathophysiology, Clinical Patterns, and Therapeutic Challenges of Intractable and Severe Forms, International Journal of Molecular Sciences. (2025) 26, no. 17, 10.3390/ijms26178538, 8538. 40943458 PMC12429651

[11] Waldman A. T. , Acute Disseminated Encephalomyelitis in Adults, UpToDate, 2024, https://www.uptodate.com/contents/acute-disseminated-encephalomyelitis-adem-in-adults#H193267825.

[12] Deschamps R. , Guillaume J. , and Ciron J. , et al.Early Maintenance Treatment Initiation and Relapse Risk Mitigation After a First Event of MOGAD in Adults: The MOGADOR2 Study, Neurology. (2024) 103, no. 3, 10.1212/WNL.0000000000209624. 38991174

[13] Sutton P. , Lutz M. W. , and Hartsell F. L. , et al.Myelin Oligodendrocyte Glycoprotein (MOG) Antibody-Associated Disease: Presentation and Outcomes of Adults at a Single Center, Journal of Neuroimmunology. (2022) 373, 10.1016/j.jneuroim.2022.577987, 577987. 36272183

